# Supplementary material for: The evolution of a counter-defense mechanism in a virus constrains its host range
Source: eLife. 2022 Aug 4;11:e79549. doi: 10.7554/eLife.79549 (PMC9391042; doi:10.7554/eLife.79549)
Supplement: Supplementary file 1. [file elife-79549-supp1.docx]

**Supplementary File 1a. Strains**

| **Bacterial Strains** | | |
| --- | --- | --- |
| **Name** | **Genotype** | **Source** |
| ML6 | MG1655 |  |
| DH5α | Cloning strain | Invitrogen |
| ML3326 | MG1655 pBAD33-*toxN* pEXT20-*toxI* | Guegler and Laub, 2021 |
| ML3328 | MG1655 pBR322-*toxIN* | Guegler and Laub, 2021 |
| ML3330 | MG1655 pBR322 | Guegler and Laub, 2021 |
| ML3772 | MG1655 pBR322-*toxIN* pKVS45 | This study |
| ML3773 | MG1655 pBR322-*toxIN* pKVS45-*dmd_T4_* | This study |
| ML3774 | MG1655 pBR322-*toxIN* pKVS45-*tifA_T4_* | This study |
| ML3775 | MG1655 pBR322-*toxIN* pKVS45-*tifA_T2_* | This study |
| ML3776 | MG1655 pBR322-*toxIN* pKVS45-*tifA_T6_* | This study |
| ML3777 | MG1655 pBR322-*toxIN* pKVS45-*tifA_RB69_* | This study |
| ML3778 | MG1655 pBAD33-*toxN* pEXT20-*tifA_T2_* | This study |
| ML3779 | MG1655 pBAD33-*toxN* pEXT20-*tifA_T4_* | This study |
| ML3780 | MG1655 pBAD33-*toxN* pEXT20-*tifA_T6_* | This study |
| ML3781 | MG1655 pBAD33-*toxN* pEXT20-*tifA_RB69_* | This study |
| ML3782 | MG1655 pBAD33-*toxN* pEXT20 | This study |
| ML3783 | MG1655 pBR322-*toxIN* pKVS45-*tifA _T4_* ΔATG | This study |
| ML3784 | MG1655 pBR322-*toxIN* pKVS45-*tifA _T4_* recoded | This study |
| ML3785 | MG1655 pBR322-*toxIN* pKVS45-*tifA_T4_* ablated toxN-site | This study |
| ML3786 | MG1655 pBR322-*toxI-toxN*-His_6_ pKVS45-*tifA_T4_*-FLAG | This study |
| ML3787 | MG1655 pBR322- *toxI-toxN*-His_6_ pKVS45-*tifA _T4_* | This study |
| ML3788 | MG1655 pBR322-*toxI-toxN* pKVS45-*tifA _T4_*-FLAG | This study |
| ML3789 | MG1655 *attB_λ_::toxIN* | This study |
| ML3790 | MG1655 *attB_λ_::toxI-toxN(K55A)* | This study |
| ML3791 | MG1655 λ-lysogen | This study |
| ML3792 | MG1655 λ-lysogen pBR322-*rIIA_T4_* pKVS45-*rIIB_T4_* | This study |
| ML3793 | *E. coli str C* | Félix d’Hérelle Reference Center for Bacterial Viruses, Université Laval |
| ML3794 | ECOR17 | Thomas S. Whittam STEC Center at Michigan State University |
| ML3795 | ECOR17 pEXT20-*ipIII_T4_* | This study |
| ML3796 | ECOR17 pEXT20-*ipIII_T4_ΔCTS* | This study |
| ML3797 | ECOR17 *ΔRM-typeI* | This study |
| ML3798 | ECOR17 *ΔRM-typeIII* | This study |
| ML3799 | ECOR17 *Δabi2* | This study |
| ML3800 | ECOR17 *Δdsr1* | This study |
| ML3801 | ECOR17 *Δhhe* | This study |
| ML3802 | ECOR17 *Δcas3* | This study |
| ML3803 | ECOR71 | Thomas S. Whittam STEC Center at Michigan State University |
| ML3804 | ECOR13 | Thomas S. Whittam STEC Center at Michigan State University |
| ML3805 | ECOR16 | Thomas S. Whittam STEC Center at Michigan State University |
| ML3806 | MG1655 pBR322-*Dsr1_ECOR17_ pKVS45* | This study |
| ML3807 | MG1655 pBR322-*Dsr1_ECOR17_ pKVS45-nrdC.5_T4_* | This study |
| ML3808 | MG1655 pCas9-*tifA_T4_*-cr4 | This study |
| ML3343 | DH5α pBAD33-*toxN* | Guegler and Laub, 2021 |
| ML3345 | DH5α pEXT20-*toxI* | Guegler and Laub, 2021 |
| ML1978 | DH5α pEXT20 | E. coli Genetic Stock Center, #12325 |
| ML3346 | DH5α pBR322-*toxIN* | Guegler and Laub, 2021 |
| ML3348 | DH5α pBR322 | Guegler and Laub, 2021 |
| ML3349 | DH5α pBR322-*toxI-toxN-*His_6_ | Guegler and Laub, 2021 |
| ML3809 | DH5α pKVS45-*dmd_T4_* | This study |
| ML3810 | DH5α pKVS45-*tifA_T4_* | This study |
| ML3811 | DH5α pKVS45-*tifA_T2_* | This study |
| ML3812 | DH5α pKVS45-*tifA_T6_* | This study |
| ML3813 | DH5α pKVS45-*tifA_RB69_* | This study |
| ML3814 | TOP10 pEXT20-*tifA_T2_* | This study |
| ML3815 | TOP10 pEXT20-*tifA_T4_* | This study |
| ML3816 | TOP10 pEXT20-*tifA_T6_* | This study |
| ML3817 | TOP10 pEXT20-*tifA_RB69_* | This study |
| ML3818 | DH5α pKVS45-*tifA_T4_* ΔATG | This study |
| ML3819 | DH5α pKVS45-*tifA _T4_* recoded | This study |
| ML3820 | DH5α pKVS45-*tifA _T4_* ablated toxN-site | This study |
| ML3821 | TOP10 pKVS45-*tifA_T4_-*FLAG | This study |
| ML3822 | DH5α pBR322-*rIIA_T4_* | This study |
| ML3823 | DH5α pKVS45-*rIIB_T4_* | This study |
| ML3824 | DH5α pEXT20-*ipIII_T4_* | This study |
| ML3825 | DH5α pEXT20-*ipIII_T4_ΔCTS* | This study |
| ML3826 | DH5α pBR322-*Dsr1_ECOR17_* | This study |
| ML3827 | DH5α pKVS45-*nrdC.5_T4_* | This study |
| ML3828 | DH5α pCas9-*tifA_T4_*-cr4 | This study |
|  | | |
| **Phage Strains** | | |
| **Name** | **Genotype** | **Source** |
| phML31 | T4 ancestor | Guegler and Laub, 2021 |
| phML32 | T4 control evo round 25 clone 1 | This study |
| phML33 | T4 evo 1 round 25 clone 1 | This study |
| phML34 | T4 evo 2 round 25 clone 1 | This study |
| phML35 | T4 evo 3 round 25 clone 1 | This study |
| phML36 | T4 evo 4 round 25 clone 1 | This study |
| phML37 | T4 evo 5 round 25 clone 3 | This study |
| phML38 | T2 | ATCC Cat #: 11303-B2 |
| phML39 | T6 | ATCC Cat #: 11303-B6 |
| phML40 | RB69 | Laval Collection, HER #  158 |
| phML41 | T4 *tifA-1* | This study |
| phML42 | T4 *tifA-2* | This study |

**Supplementary File 1b. Plasmids**

| **Plasmid** | **Description** | **Source** |
| --- | --- | --- |
| pBR322 empty vector | Derivative of pBR322 with pTet removed | Guegler and Laub, 2021 |
| pBR322-*toxIN* | Full *toxIN* locus | Guegler and Laub, 2021 |
| pBR322-*toxI-toxN-*His_6_ | *toxIN* locus with C-terminal His_6_-tagged ToxN | Guegler and Laub, 2021 |
| pBR322-*rIIA_T4_* | *rIIA* under native T4 middle promoter | This study |
| pBR322-*Dsr1_ECOR17_* | *Dsr1* from ECOR17 under native promoter region | This study |
| pKVS45 | aTc inducible vector |  |
| pKVS45-*tifA _T4_* | aTc inducible TifA expression | This study |
| pKVS45-*dmd _T4_* | aTc inducible Dmd expression | This study |
| pKVS45-*tifA _T2_* | aTc inducible TifA expression | This study |
| pKVS45-*tifA _T6_* | aTc inducible TifA expression | This study |
| pKVS45-*tifA _RB69_* | aTc inducible TifA expression | This study |
| pKVS45-*tifA_T4_* ΔATG | aTc inducible TifA ΔATG expression | This study |
| pKVS45-*tifA _T4_* recoded | aTc inducible TifA recoded expression | This study |
| pKVS45-*tifA_T4_* ablated toxN-site | aTc inducible TifA ablated toxN-site expression | This study |
| pKVS45-*tifA_T4_*-FLAG | aTc inducible TifA with C-terminal FLAG tag | This study |
| pKVS45-*rIIB_T4_* | aTc inducible *rIIA* expression | This study |
| pKVS45-*nrdC.5_T4_* | aTc inducible *nrdC.5* expression | This study |
| pBAD33-*toxN* | Arabinose inducible ToxN expression | Guegler and Laub, 2021 |
| pEXT20 | IPTG inducible vector | E. coli Genetic Stock Center, #12325 |
| pEXT20-*toxI* | IPTG inducible *toxI* expression | Guegler and Laub, 2021 |
| pEXT20-*tifA_T2_* | IPTG inducible *tifA_T2_* expression | This study |
| pEXT20-*tifA_T4_* | IPTG inducible *tifA_T4_* expression | This study |
| pEXT20-*tifA_T6_* | IPTG inducible *tifA_T6_* expression | This study |
| pEXT20-*tifA_RB69_* | IPTG inducible *tifA_RB69_* expression | This study |
| pEXT20-*ipIII_T4_* | IPTG inducible *ipIII_T4_* expression | This study |
| pEXT20-*ipIII_T4_ΔCTS* | IPTG inducible *ipIII_T4_ΔCTS* expression | This study |
| pCas9 | Cas9 with restriction site to clone guideDNA | Addgene #42876 |
| pCas9-*tifA_T4_*-cr4 | Cas9 with guide targeting *tifA_T4_* | This study |
| pKD46 | L-ara inducible λ-red recombinase | Datsenko and Wanner, 2000 *E. coli* stock center CGSC# 7669 |
| pKD4 | Kanamycin resistance cassette | Datsenko and Wanner, 2000 *E. coli* stock center CGSC# 7632 |

**Supplementary File 1c. Primers**

| Name | Purpose | Sequence |
| --- | --- | --- |
| SS-1 | Primer to amplify *tifA* locus (forward) | ATAAAGCATTATTCACCTACCACTTCAGCG |
| SS-2 | Primer to amplify *tifA* locus (reverse) | GAGCTAGCAGCTGCTGAACAACAACT |
| SS-3 | Divergent primer to amplify *tifA* segmental amplification (forward) | TTCCTGACGTTATACGGAGTAGG |
| SS-4 | Divergent primer to amplify *tifA* segmental amplification (reverse) | TTCCTGACGTTATACGGAGTAGG |
| SS-5 | Construct pKVS45-*tifA* (forward) | cgaattcgagctcggtacccATGCATATTGTTTTATTTAAACCTAC |
| SS-6 | Construct pKVS45-*tifA* (reverse) | ggtcgactctagaggatccccTTATTTTAAAATTTCTGCGTAATCAC |
| SS-7 | Construct pKVS45-*dmd* (forward) | cgaattcgagctcggtacccATGGAATTGGTAAAGGTAGT |
| SS-8 | Construct pKVS45-*dmd* (reverse) | ggtcgactctagaggatccccTTATCCTCGGCAATCCACTT |
| SS-9 | Construct pKVS45-*tifA*-FLAG (forward) | ggggatcctctagagtcgacc |
| SS-10 | Construct pKVS45-*tifA*-FLAG (reverse) | TTActtgtcatcgtcgtccttgtagtcGCTTCCGCTTCCTTTTAAAATTTCTGCGTAATCACATGTTACAAACTGTTTC |
| SS-11 | Construct pEXT20-*tifA_T2_* (forward) | AGGTTTCTCCATACAGGAGGTACCCATGCATATTGTTTTATTTAAACCTACTC |
| SS-12 | Construct pEXT20-*tifA_T2_* (reverse) | TGCAGGTCGACTCTAGAGGATCCCCTTATTTTAAAACTTTTGCGTAATCAC |
| SS-13 | Construct pEXT20-*tifA_T6_* (forward) | AGGTTTCTCCATACAGGAGGTACCCATGCATATTGTTTTATTTAAACCTACTC |
| SS-14 | Construct pEXT20-*tifA_T6_* (reverse) | TGCAGGTCGACTCTAGAGGATCCCCCTATTTTAAAACTTTTGCATAATCAC |
| SS-15 | Construct pEXT20-*tifA_RB69_* (forward) | AGGTTTCTCCATACAGGAGGTACCCATGTATTCAACTGTGTTTAAACCATC |
| SS-16 | Construct pEXT20-*tifA_RB69_* (reverse) | TGCAGGTCGACTCTAGAGGATCCCCCTATTTTAAACTTTTGCGAAATTTG |
| SS-17 | Construct pEXT20-*tifA_T2_* (forward) | CACACAGGAAACAGAATTCGAGCTCATGCATATTGTTTTATTTAAACCTACTCC |
| SS-18 | Construct pEXT20-*tifA_T2_* (reverse) | GAAGCTTGCATGCCTGCAGGTCGACTTATTTTAAAACTTTTGCGTAATCACATG |
| SS-19 | Construct pEXT20-*tifA_T4_* (forward) | CACACAGGAAACAGAATTCGAGCTCATGCATATTGTTTTATTTAAACCTACTC |
| SS-20 | Construct pEXT20-*tifA_T4_* (reverse) | GAAGCTTGCATGCCTGCAGGTCGACTTATTTTAAAATTTCTGCGTAATCACATG |
| SS-21 | Construct pEXT20-*tifA_T6_* (forward) | CACACAGGAAACAGAATTCGAGCTCATGCATATTGTTTTATTTAAACCTACTCC |
| SS-22 | Construct pEXT20-*tifA_T6_* (reverse) | GAAGCTTGCATGCCTGCAGGTCGACCTATTTTAAAACTTTTGCATAATCACATG |
| SS-23 | Construct pEXT20-*tifA_RB69_* (forward) | CACACAGGAAACAGAATTCGAGCTCATGTATTCAACTGTGTTTAAACCATC |
| SS-24 | Construct pEXT20-*tifA_RB69_* (reverse) | GAAGCTTGCATGCCTGCAGGTCGACCTATTTTAAACTTTTGCGAAATTTGCG |
| SS-25 | Construct pAH150-*toxIN* (*toxIN* forward) | GCGAGAGTAGGGAACTGCCAGGCATTTATGGCCGCGTTTATCTCATTCCACG |
| SS-26 | Construct pAH150-*toxIN* (*toxIN* reverse) | cgactctagaggatccccggGTACCCTTATATTGGATGAGAGCAAAAAAAATAGGTCC |
| SS-27 | Construct pAH150-*toxIN* (backbone forward) | GGTACccggggatcctctagagtcg |
| SS-28 | Construct pAH150-*toxIN* (backbone reverse) | ATGCCTGGCAGTTCCCTACTC |
| SS-29 | Construct pAH150-*toxIN(K55A)* (forward) | GCGGCATGGCATGCTAATGTAAAAGAGTCATC |
| SS-30 | Construct pAH150-*toxIN(K55A)* (reverse) | TGGCGATGTTAAAGGTGCTAAATATTTATGTCC |
| SS-31 | Construct pKVS45-*tifA* ΔATG (forward) | CATATTGTTTTATTTAAACCTACTCCG |
| SS-32 | Construct pKVS45-*tifA* ΔATG (reverse) | GGGTACCTCCTGTATGGAGAAACCTAGG |
| SS-33 | Construct pKVS45-*tifA* ΔtoxN-site (forward) | ATTACGCAGAGATTTTAAAATAAGGGGATCCTCTAGAG |
| SS-34 | Construct pKVS45-*tifA* ΔtoxN-site (reverse) | ATTTTAAAATCTCTGCGTAATCACATGTTACAAACTG |
| SS-35 | Construct pEXT20-*ipIII_T4_* (forward) | CACACAGGAAACAGAATTCGAGCTCATGAAAACATATCAAGAATTTATTGCCG |
| SS-36 | Construct pEXT20-*ipIII_T4_* (reverse) | GAAGCTTGCATGCCTGCAGGTCGACTTAAGAATTACCACGGGCTGCATTAG |
| SS-37 | Construct pBR322-*rIIA_T4_* (forward) | CTTTCGTCTTCAAGAATTCTCATGTTCGATATGGGAGAAGCCGAAG |
| SS-38 | Construct pBR322-*rIIA_T4_* (reverse) | CAAGAATTGATTGGCTCCAATTCTTTTATTTAAATTGTTCAGTAACGTCTTCAAC |
| SS-39 | Amplify pBR322 vector (forward) | AAGAATTGGAGCCAATCAATTCTTG |
| SS-40 | Amplify pBR322 vector (reverse) | ACATGAGAATTCTTGAAGACGAAAG |
| SS-41 | Construct pKVS45-*rIIB_T4_* (forward) | CGAATTCGAGCTCGGTACCCATGTACAATATTAAATGCCTGAC |
| SS-42 | Construct pKVS45-*rIIB_T4_* (reverse) | GGTCGACTCTAGAGGATCCCCTTATTTAAATTGTTCAGTAACGTCT |
| SS-43 | Construct pBR322-*Dsr1_ECOR17_* (forward) | CTTTCGTCTTCAAGAATTCTCATGTAGGTGTATGGCAAGTTTATGACAAGAGT |
| SS-44 | Construct pBR322- *Dsr1_ECOR17_* (reverse) | CAAGAATTGATTGGCTCCAATTCTTTCACACGCTGCGCCTT |
| SS-45 | Construct pKVS45-*nrdC.5_T4_* (forward) | CGAATTCGAGCTCGGTACCCATGAAAACTCGTTCTCAAATTG |
| SS-46 | Construct pKVS45-*nrdC.5_T4_* (reverse) | GGTCGACTCTAGAGGATCCCCCTAGTTCAGTGCATTTAGTGC |
| SS-47 | Construct pCas9-61.4-cr4 (forward) | AAACAATTCCACTCGACCAAATGGG |
| SS-48 | Construct pCas9-61.4-cr4 (reverse) | AAAACCCATTTGGTCGAGTGGAATT |

**Supplementary File 1d. Antibodies**

| Reagent | Source | Catalogue Number | RRID |
| --- | --- | --- | --- |
| Anti-FLAG M2 magnetic beads | Sigma | M8823 | AB_2637089 |
| Recombinant anti-6X His tag rabbit antibody | Abcam | AB200537 |  |
| DYKDDDDK tag rabbit mAb | Cell Signaling Technology | 14793 | AB_2572291 |
| Goat anti-rabbit IgG (H+L) secondary antibody, HRP | ThermoFisher Scientific | 32460 | AB_1185567 |
