# Supplementary material for: The evolution of a counter-defense mechanism in a virus constrains its host range
Source: eLife. 2022 Aug 4;11:e79549. doi: 10.7554/eLife.79549 (PMC9391042; doi:10.7554/eLife.79549)
Supplement: Source data 1. [file elife-79549-data1.zip › source-data/gels/Figures_source-data_labelled-gels_D03.pdf]

The evolutions of a counter-defense mechanism in a virus constrains its host range

Srikant S, Guegler CK, Laub MT

Labelled source data

Figure 1E
source data file: Figure1_source-data1.tif

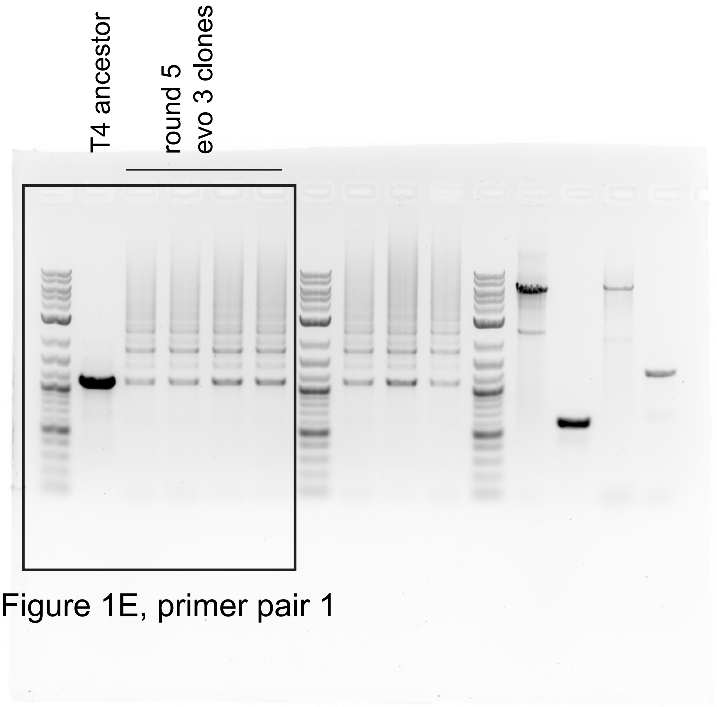

Figure 1E, primer pair 1

Figure 1E
source data file: Figure1_source-data2.tif

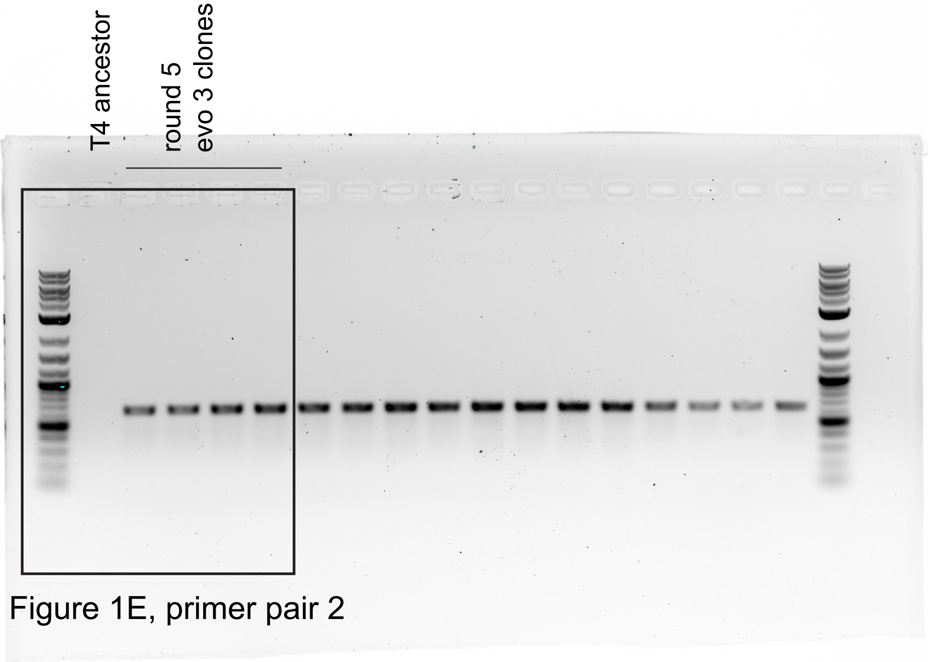

Figure 1E, primer pair 2

Figure 2E: anti-His_6 blot
Source data file: Figure2_source-data1-antiHis6blot_chemiluminescence.png

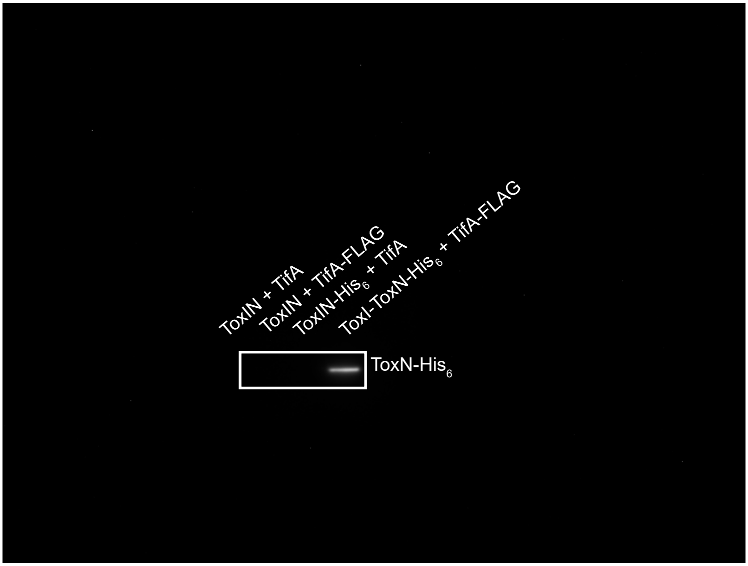

Figure 2E: anti-FLAG blot
Source data file: Figure2_source-data2-antiFLAGblot_chemiluminescence.png

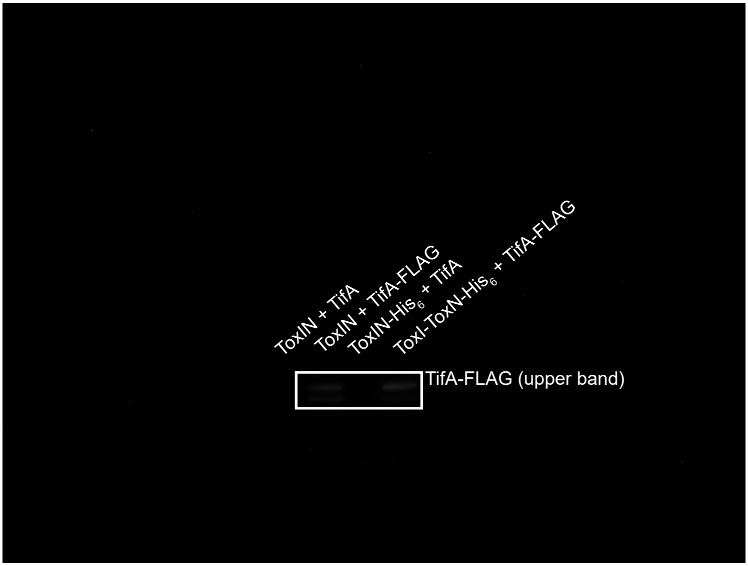

Image with contrast adjusted to better show bands:

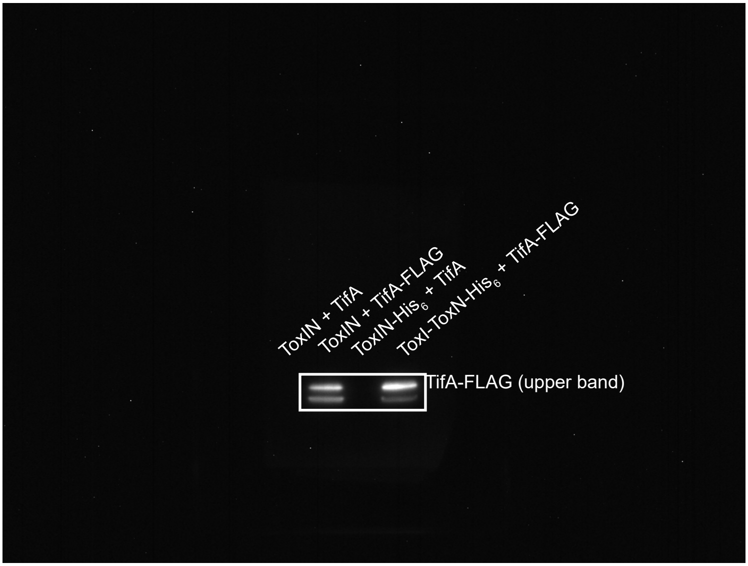

Figure 4E
source data file: Figure4_source-data1.png

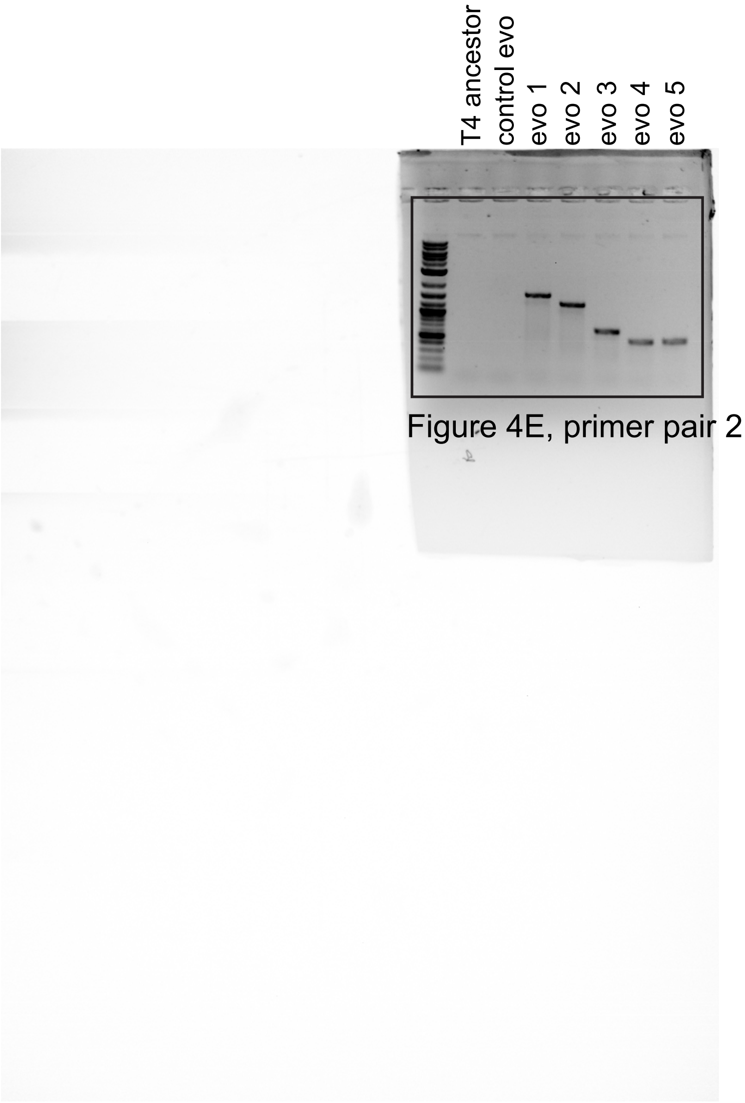

Figure 4 - figure supplement 2A
source data file: Figure4_figure-supplement2A_source-data1.tif

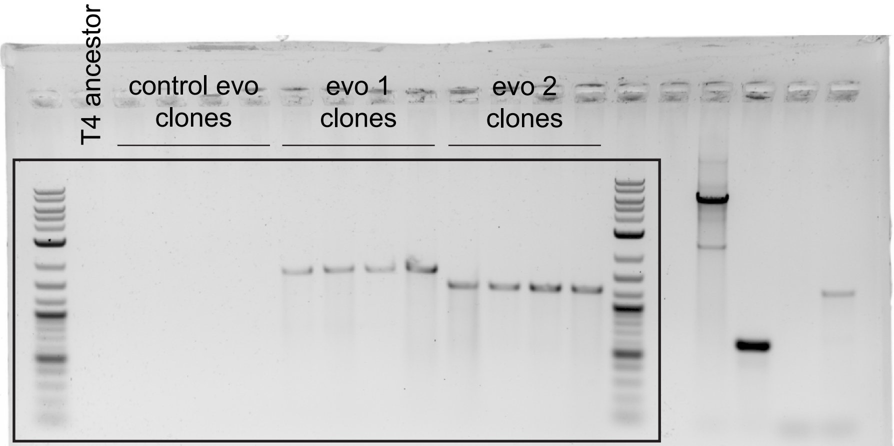

Figure 4-figure supplement 2A, primer pair 2

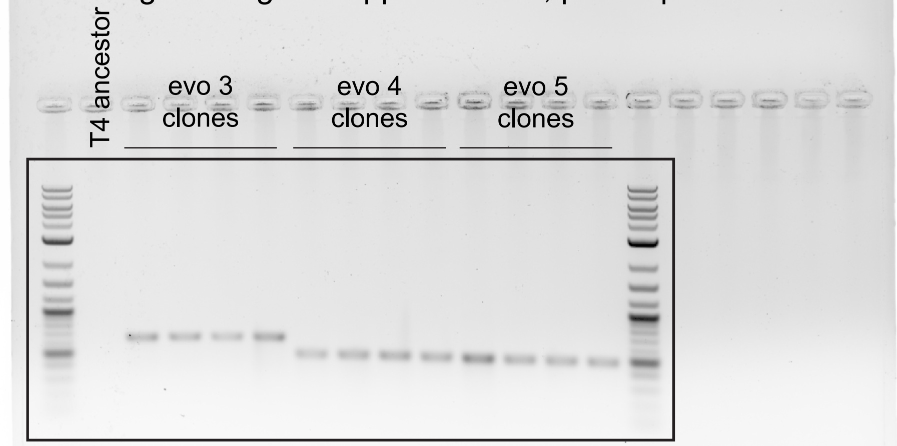

Figure 4-figure supplement 2A, primer pair 2

Figure 5 - figure supplement 1A
source data file: Figure5_figure-supplement1A_source-data1.tif

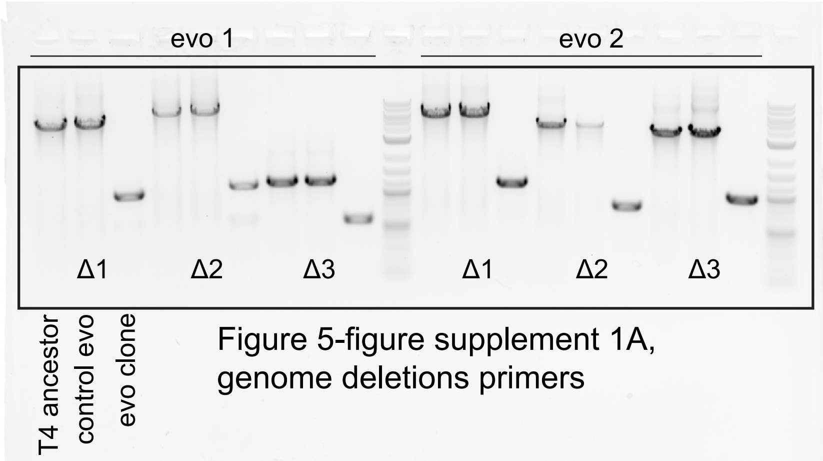

Figure 5-figure supplement 1A, genome deletions primers

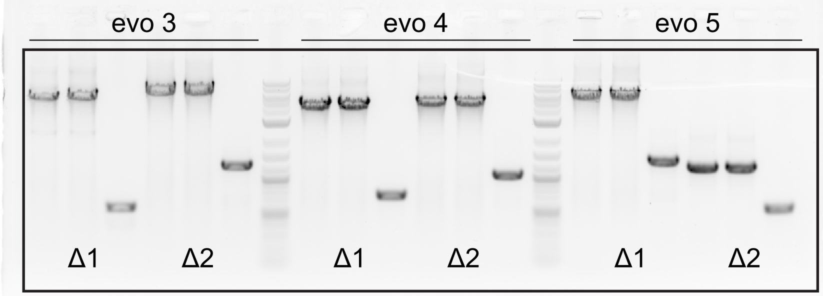

Figure 5-figure supplement 1A, genome deletions primers

Figure 5 - figure supplement 1C
source data file: Figure5_figure-supplement1C_source-data1.tif

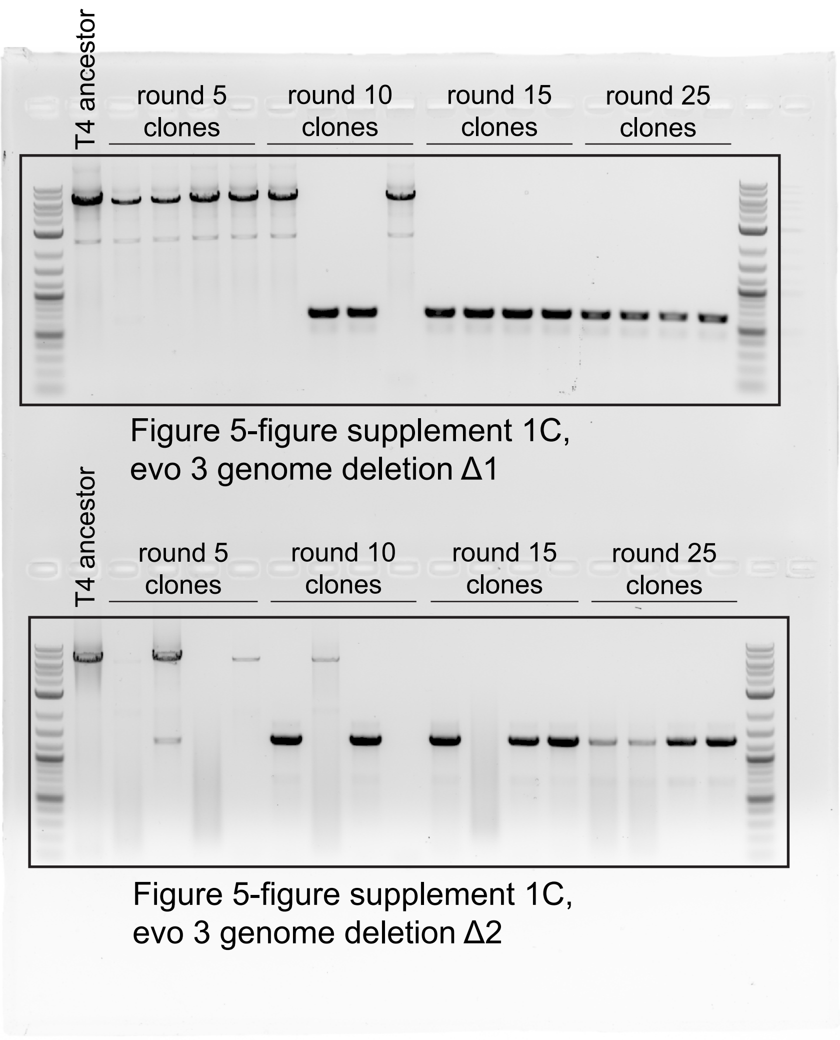

Figure 5-figure supplement 1C,
evo 3 genome deletion Δ1

Figure 5-figure supplement 1C,
evo 3 genome deletion Δ2

Figure 5 - figure supplement 1C
source data file: Figure5_figure-supplement1C_source-data2.tif

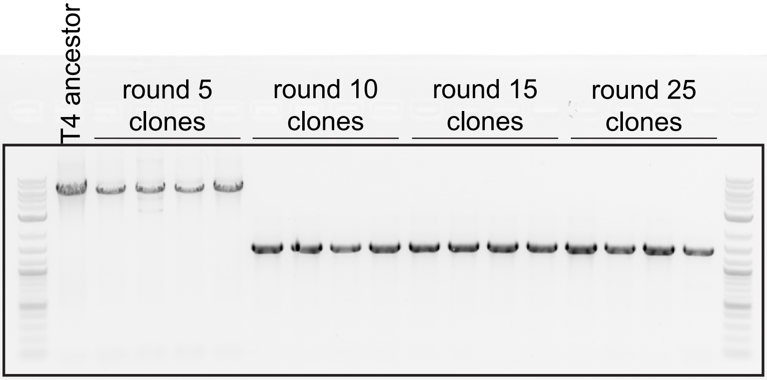

Figure 5-figure supplement 1C,
evo 5 genome deletion Δ1

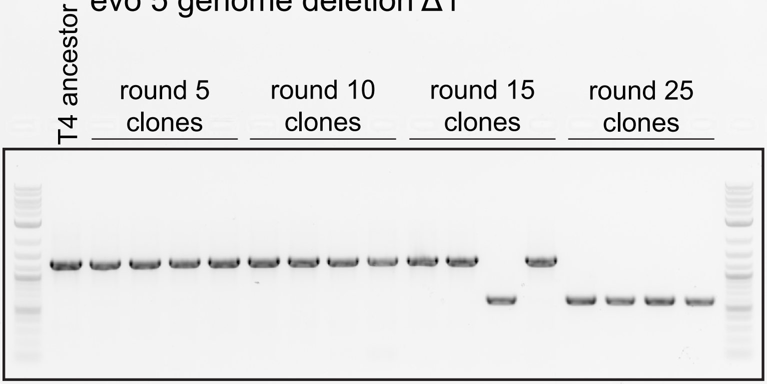

Figure 5-figure supplement 1C,
evo 5 genome deletion Δ2
