# Supplementary figures and images for: The evolution of a counter-defense mechanism in a virus constrains its host range
Source: eLife. 2022 Aug 4;11:e79549. doi: 10.7554/eLife.79549 (PMC9391042; doi:10.7554/eLife.79549)

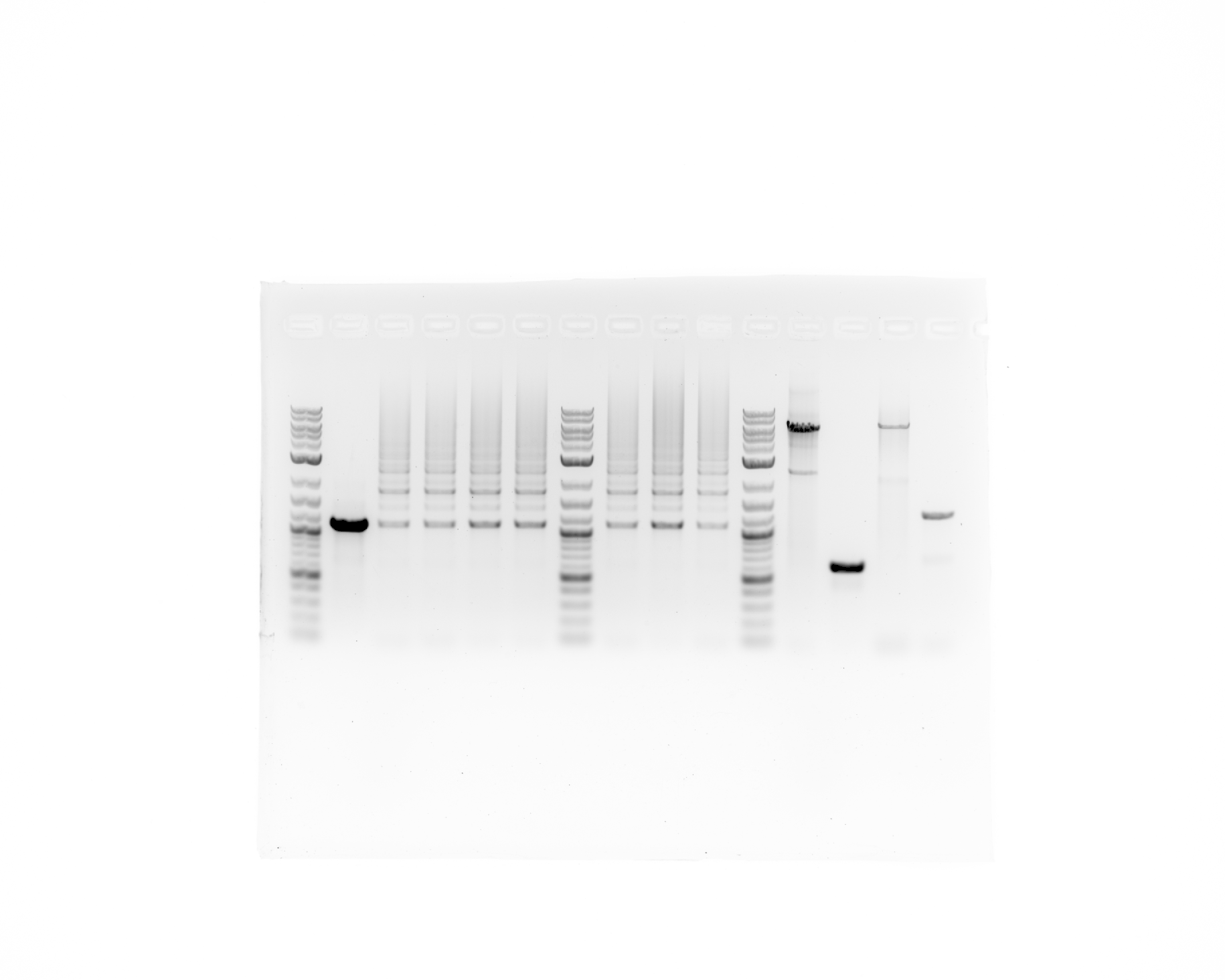

Supplement: Source data 1. [file elife-79549-data1.zip › source-data/gels/Figure1_source-data1.tif]

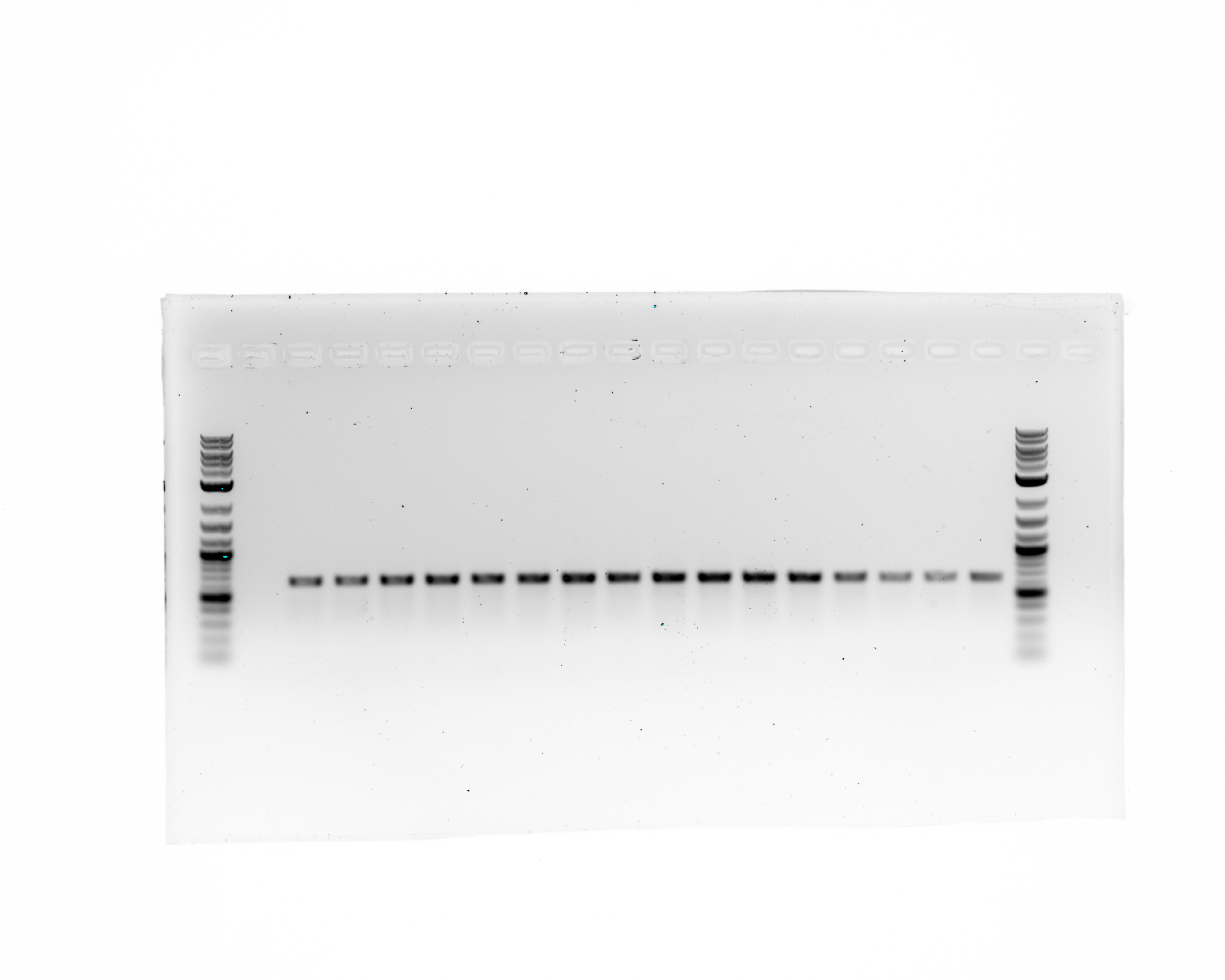

Supplement: Source data 1. [file elife-79549-data1.zip › source-data/gels/Figure1_source-data2.tif]

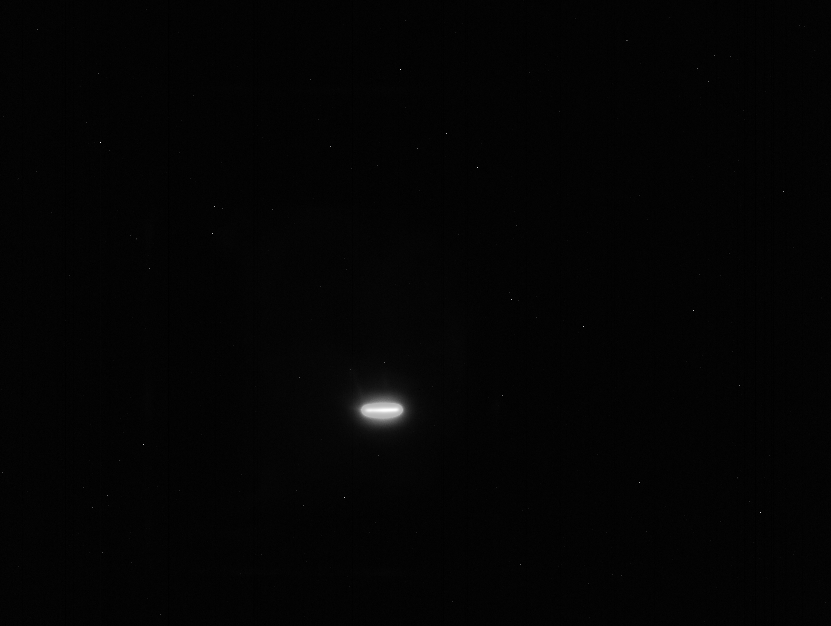

Supplement: Source data 1. [file elife-79549-data1.zip › source-data/gels/FIgure2_source-data1-antiHis6blot_chemiluminescence.png]

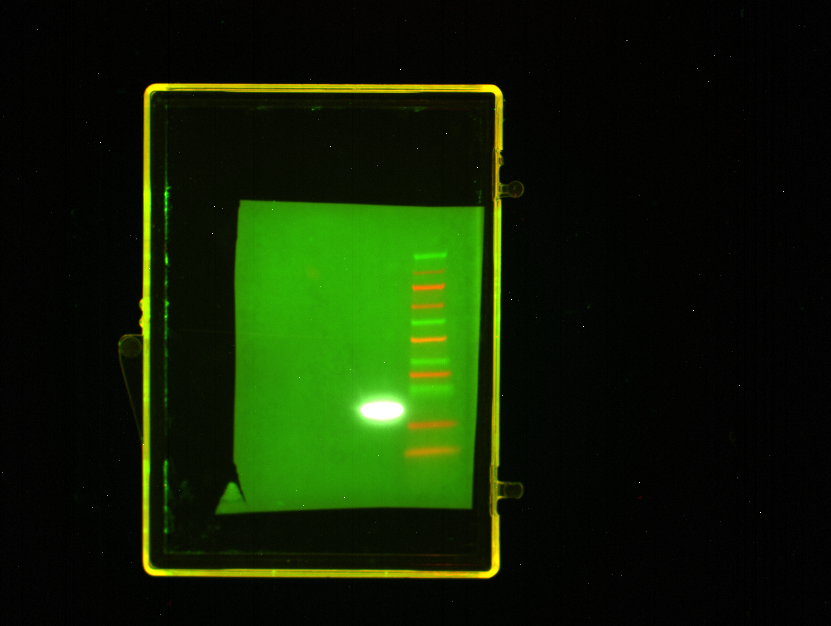

Supplement: Source data 1. [file elife-79549-data1.zip › source-data/gels/FIgure2_source-data1-antiHis6blot_chemiluminescenceandfluorescentladder_overlaid.png]

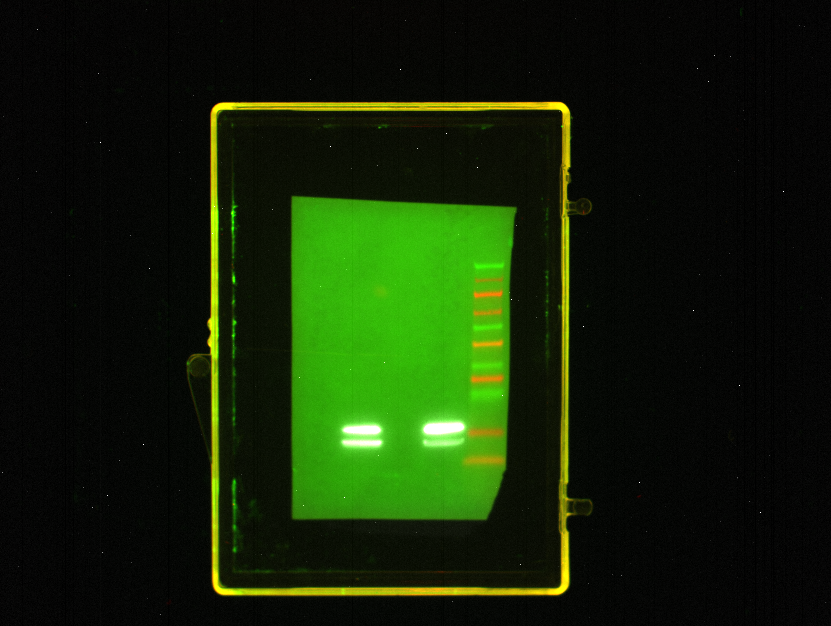

Supplement: Source data 1. [file elife-79549-data1.zip › source-data/gels/FIgure2_source-data2-antiFLAGblot_chemilumienescenceandfluorescentladder_overlaid.png]

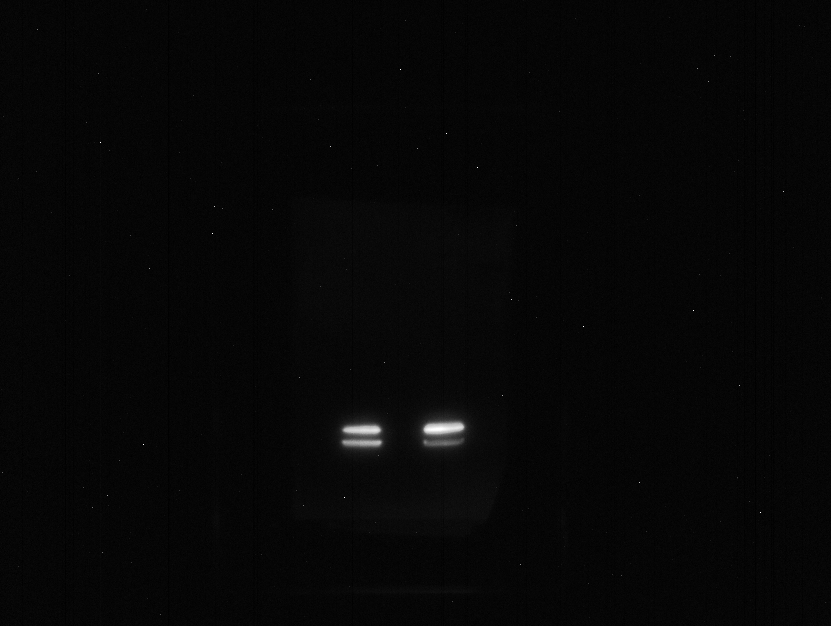

Supplement: Source data 1. [file elife-79549-data1.zip › source-data/gels/FIgure2_source-data2-antiFLAGblot_chemiluminescence.png]

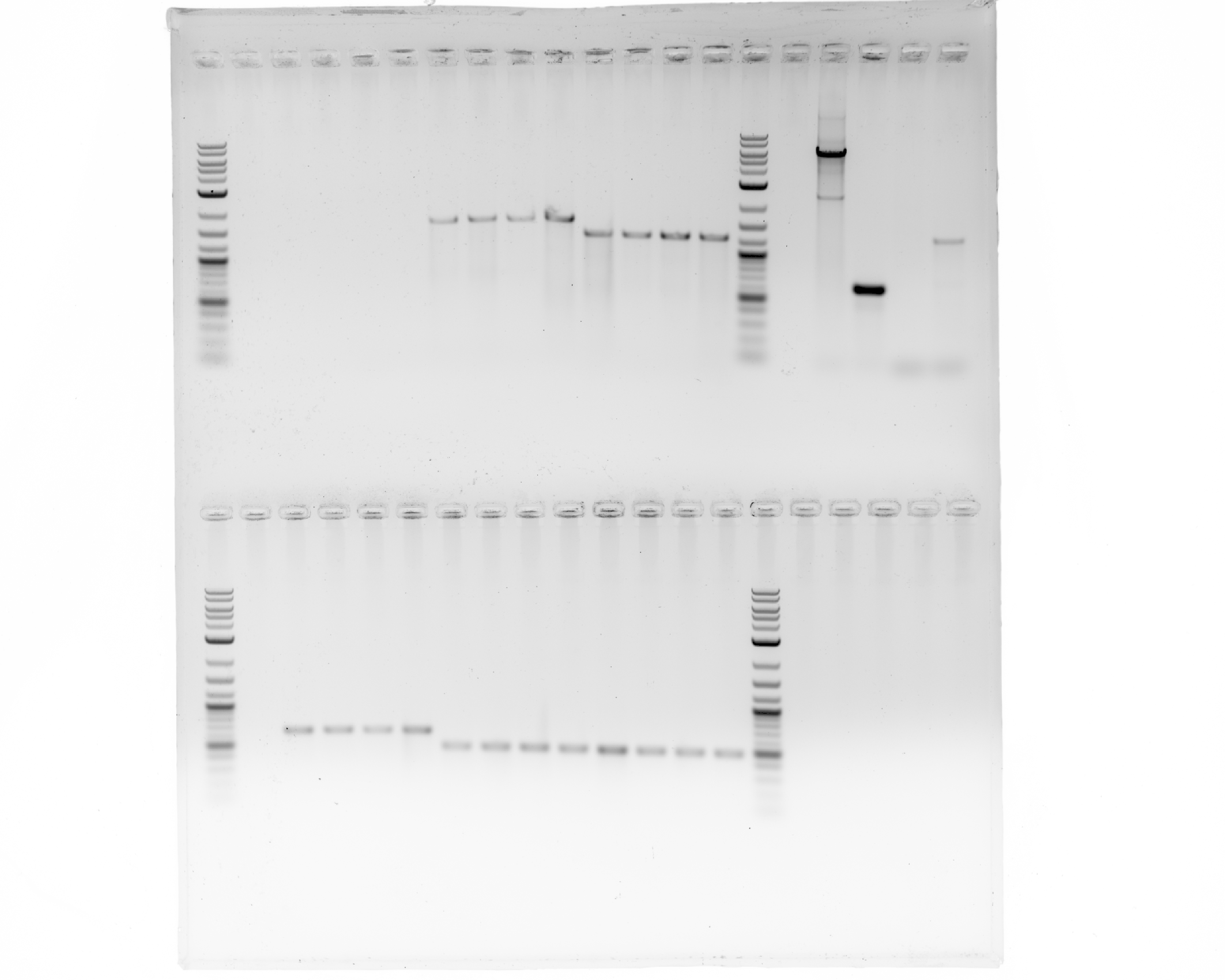

Supplement: Source data 1. [file elife-79549-data1.zip › source-data/gels/Figure4_figure-supplement2A_source-data1.tif]

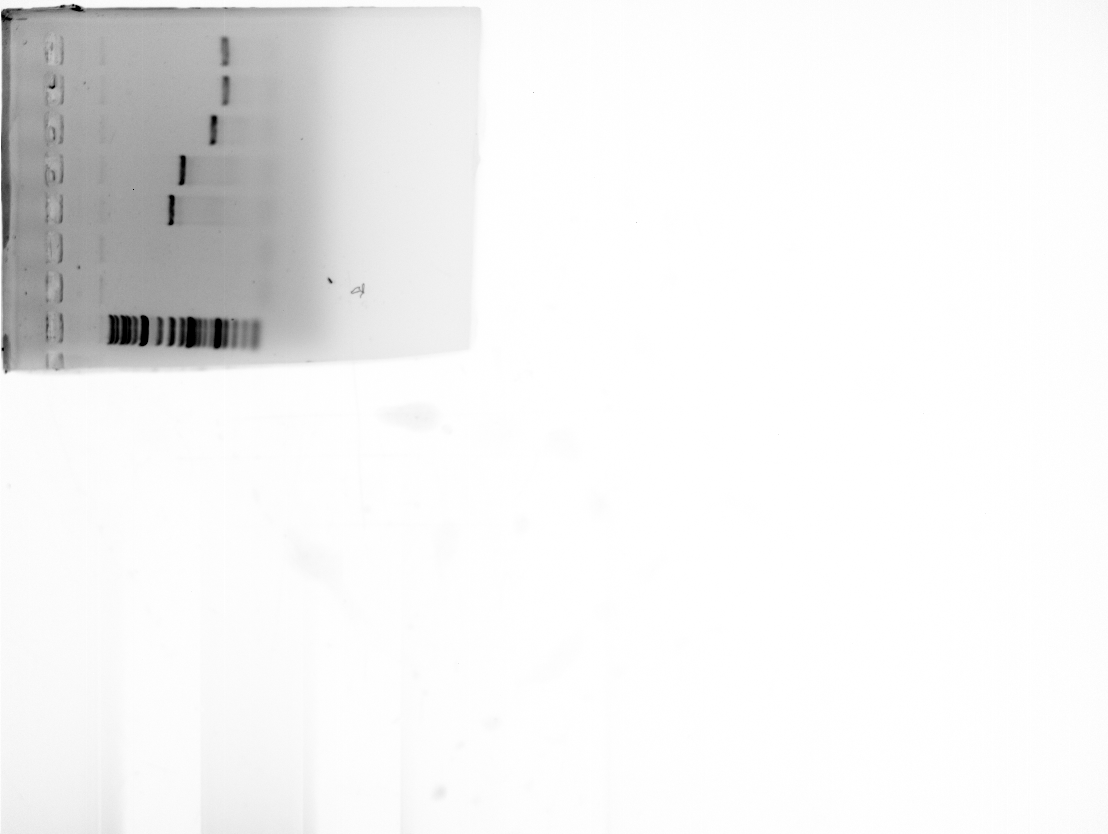

Supplement: Source data 1. [file elife-79549-data1.zip › source-data/gels/Figure4_source-data1.png]

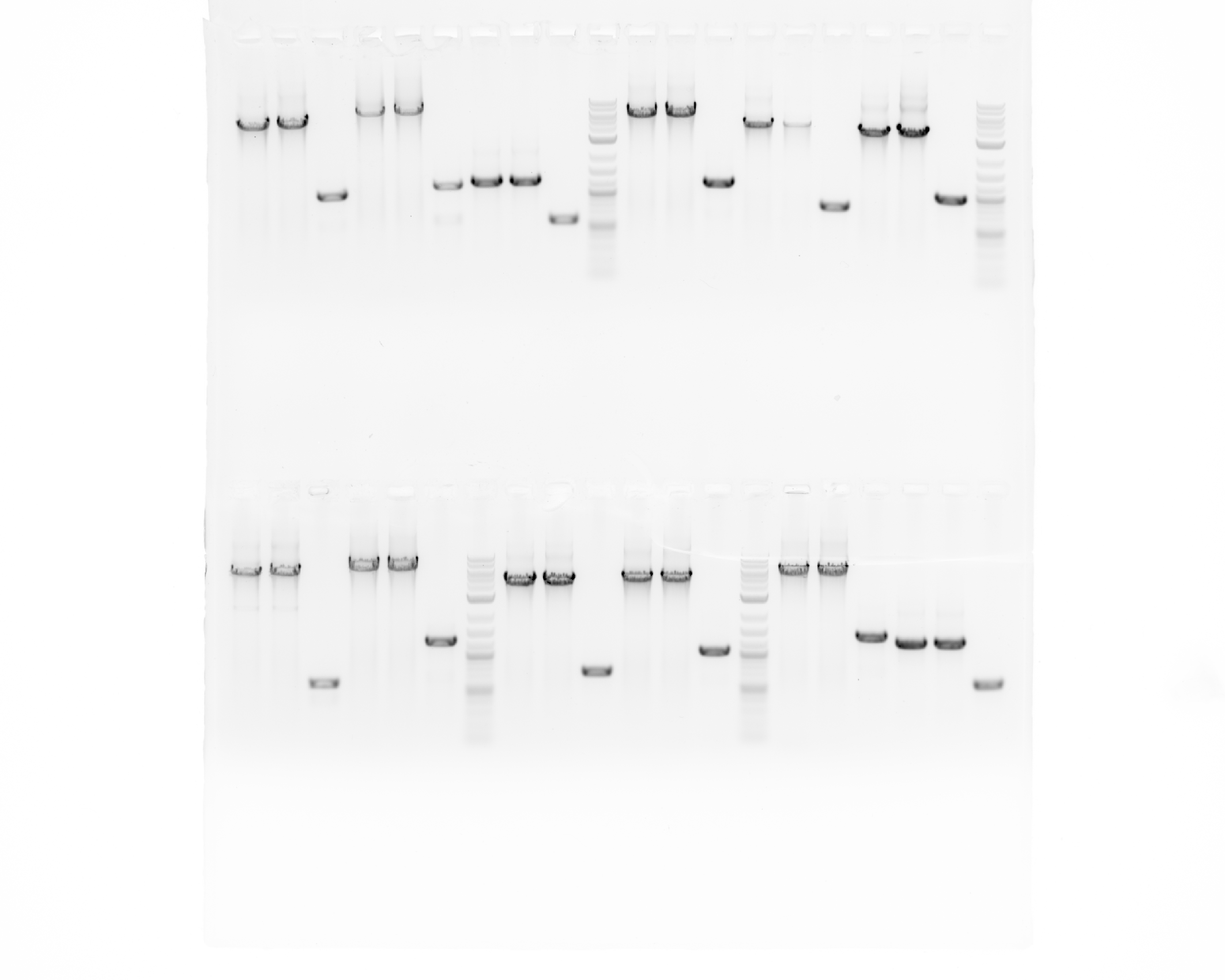

Supplement: Source data 1. [file elife-79549-data1.zip › source-data/gels/Figure5_figure-supplement1A_source-data1.tif]

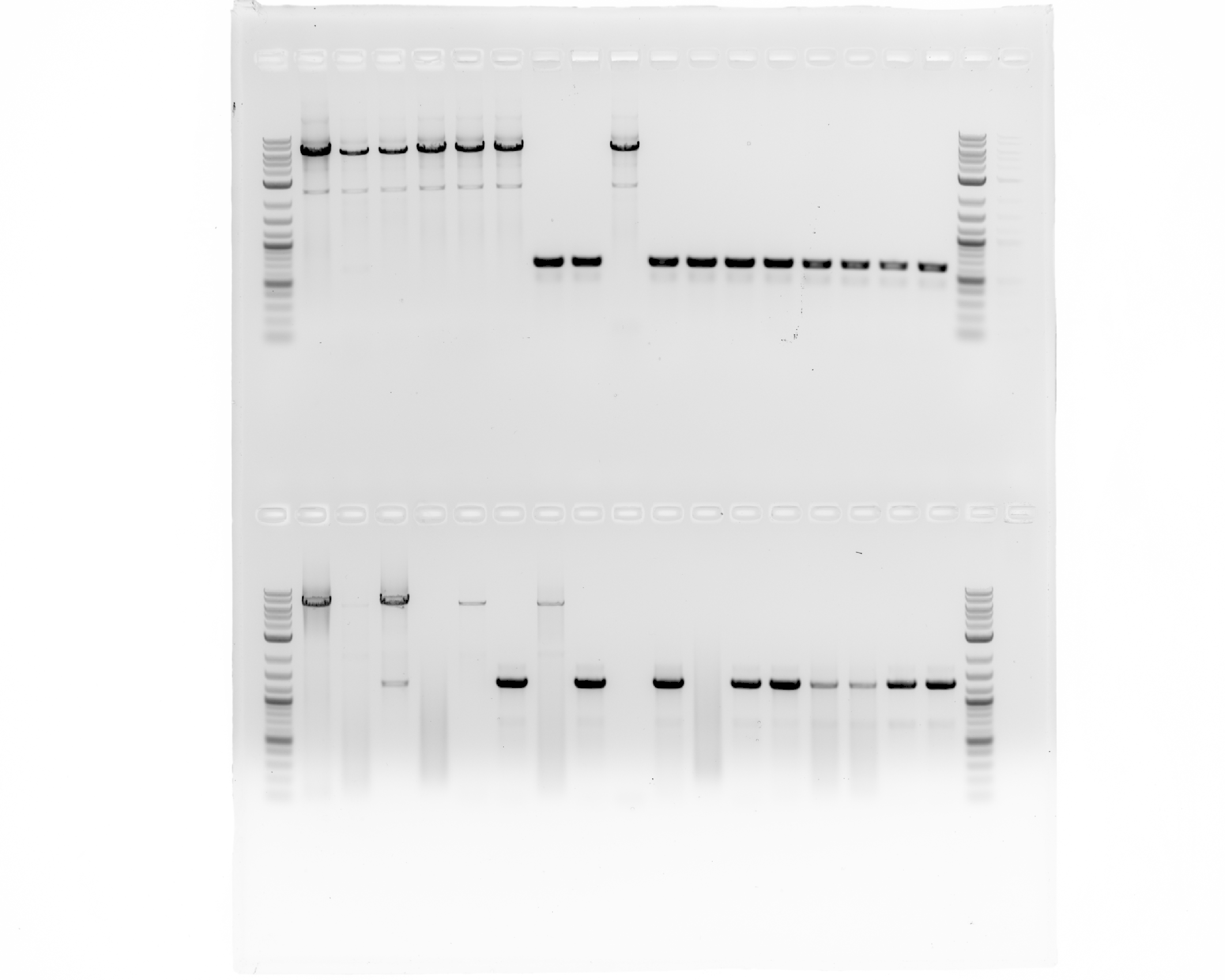

Supplement: Source data 1. [file elife-79549-data1.zip › source-data/gels/Figure5_figure-supplement1C_source-data1.tif]

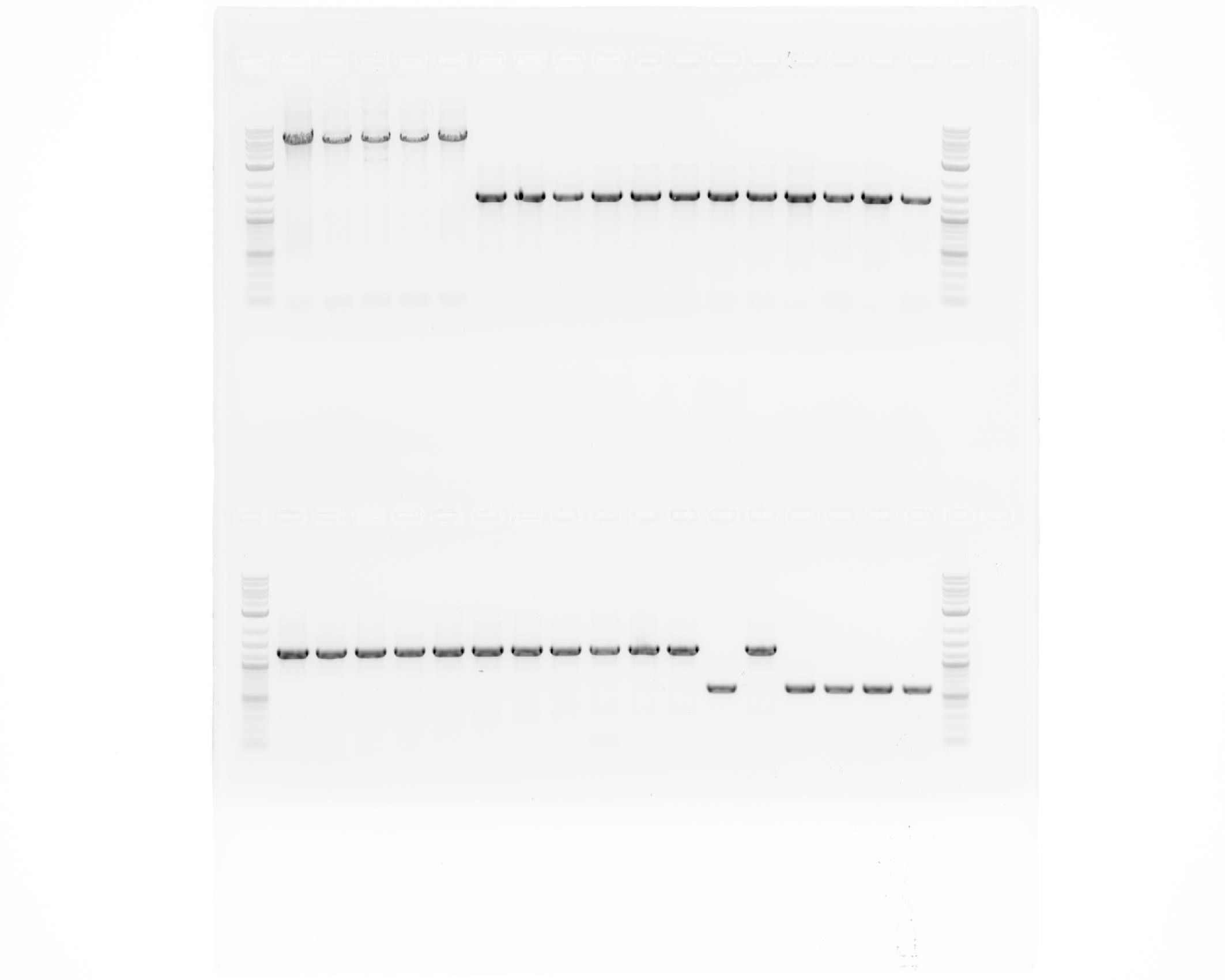

Supplement: Source data 1. [file elife-79549-data1.zip › source-data/gels/Figure5_figure-supplement1C_source-data2.tif]
